# Contrast sensitivity deficits in patients with mutation-proven inherited retinal degenerations

**DOI:** 10.1186/s12886-018-0982-0

**Published:** 2018-12-07

**Authors:** Badr O. Alahmadi, Amro A. Omari, Maria Fernanda Abalem, Chris Andrews, Dana Schlegel, Kari H. Branham, Naheed W. Khan, Abigail Fahim, Thiran Jayasundera

**Affiliations:** Department of Ophthalmology and Visual Sciences, W. K. Kellogg Eye Center, University of Michigan, Ann Arbor, MI 48150 USA

**Keywords:** Retinal dystrophy, Retinitis Pigmentosa, Stargardt disease, Best disease, Contrast sensitivity

## Abstract

**Background:**

Patients with retinal diseases frequently complain of poor visual function even when visual acuity is relatively unaffected. This clinical finding has been attributed to deficits in contrast sensitivity (CS). The purpose of our study was to evaluate the CS in patients with clinical and genetic diagnosis of inherited retinal degeneration (IRD) and relatively preserved visual acuity.

**Methods:**

Seventeen patients (30 eyes) with IRD and visual acuity of 20/40 or better, and 18 controls (18 eyes) without any ocular condition underwent slit lamp examination, visual acuity testing via standard Snellen chart testing, CS testing via the Quick Contrast Sensitivity Function (QCSF), and Spectral Domain Optical Coherence Tomography (SD-OCT). CS were measured at 1.0, 1.5, 3.0, 6.0, 12.0, and 18.0 cycles per degree (cpd). T tests with general estimated equations were used to compare CS between groups. Wald chi square followed by pairwise comparisons was used to compare CS between multiple groups.

**Results:**

We included 12 patients with rod-cone dystrophy (RCD), 3 patients with Stargardt disease (STGD) and 2 patients with Best disease. Patients with IRD had significantly worse CS than controls (*p* < 0.001) in all spatial frequencies. Patients with STGD had more marked deficits in CS than patients with Best disease (*p* < 0.001) and RCD (*p* < 0.001) despite having similar visual acuities.

**Conclusion:**

Patients with IRD, especially patients with STGD with relatively preserved visual acuity have marked deficits in CS when measured across a range of spatial frequencies. We recommend that clinical trials for STGD incorporate CS measured over a range of spatial frequencies as a secondary clinical endpoint for monitoring visual function. CS may provide an explanation for complaints of visual dysfunction when visual acuity is not significantly altered.

## Background

Patients with inherited retinal degeneration (IRD) often complain of poor central vision, despite having a visual acuity near 20/20 [1–3]. One possible explanation could be deficiencies in contrast sensitivity (CS) in these patients. However, there are a number of ongoing clinical trials for patients with IRD where the primary outcome measure is visual acuity improvement or stability [4–6]. Studies on different ophthalmic conditions have found deficits in CS when visual acuity is normal or near normal [7–9], and CS testing provides an alternate method of testing visual function [10–12]. Contrast sensitivity deficits have been described for patients with retinitis pigmentosa [1, 13–19]. Others such as Yioti et al. described structural changes observed on Spectral Domain Optical Coherence Tomography (SD-OCT) corresponding to deficits of CS in patients with retinitis pigmentosa [15].

Contrast sensitivity is typically measured using the Pelli-Robson charts, which only allow CS to be measured at a limited number of spatial frequencies and therefore may underestimate CS deficits. Although previous studies that evaluated CS at different spatial frequencies in patients with retinitis pigmentosa have found reductions at low and high spatial frequencies, CS deficits were more likely to be found at the higher spatial frequencies [1, 13, 14, 19, 20]. Nevertheless, these studies have been limited to patients clinically diagnosed with retinitis pigmentosa. Although previous studies have looked at both chromatic and achromatic CS in Stargardt disease (STGD) [21, 22], there is a paucity of work to describe CS deficits across different spatial frequencies in patients with macular dystrophies, such as STGD and Best disease.

With the advent of new methods such as the Quick Contrast Sensitivity Function (QCSF) that provide a more comprehensive evaluation of CS losses at a greater number of contrast and spatial frequency combinations [23], this study aimed to compare CS deficits among patients with both a clinical and a genetic diagnosis of an IRD and normal controls who presented with VA acuities better than 20/40.

## Methods

This study was conducted at the W. K. Kellogg Eye Center, University of Michigan. The protocol design and conduct adhered to the tenets of the Declaration of Helsinki. The study was approved by the University of Michigan Medical School’s Internal Review Board (HUM 12099). Patients were recruited from the Kellogg Eye Center Inherited Retinal Degeneration Clinic between January and October of 2017. Normal control patients were recruited as volunteers from the University of Michigan’s Health Research Website. Written informed consent was obtained from all subjects before participation in the study.

Inclusion criteria for the study were that patients had a clinical and a genetic diagnosis of an IRD and best-corrected visual acuity (BCVA) of 20/40 or better. Patients were excluded if they presented with a refractive error exceeding ±6.00 diopter (D) spherical equivalent, more than 2 diopters of keratometric astigmatism, had opacity of the ocular media (nuclear, cortical, or posterior subcapsular lens opacity) that is greater than grade 1 according to the Lens Opacities Classification System III system [24]; had a history of any ocular or retinal diseases that could affect vision other than the IRD (except for cystoid macular edema), or any systemic or neurological disease that could affect vision, had intraocular surgery in the past 3 months, and were unable to provide informed consent. Inclusion and exclusion criteria for control participants were the same except that they had no known condition that could affect vision. Patients younger than 18 years old were not included.

All patients underwent a comprehensive routine ocular examination, which was performed by a retina specialist. This included biomicroscopy of anterior and posterior segments, applanation tonometry, visual acuity (VA) using a Snellen chart, and a dilated fundus examination with a 78 diopter non-contact lens (Volk ®). Spectral Domain Optical Coherence Tomography (SD-OCT, Spectralis, Heidelberg Engineering, Heidelberg, Germany) was performed to evaluate the presence of cystoid macular edema (CME). Demographic variables, including age, race, sex, and medical conditions were also recorded.

Contrast sensitivity testing was assessed by the Quick Contrast Sensitivity Function (QCSF) on the AST Sentio platform (Adaptive Sensory Technology, United States). The QCSF is a prototype of a quick contrast sensitivity testing device that, unlike the standard Pelli-Robson chart, allows the examiner to test CS over a wider variety of contrast levels and spatial frequency combinations [7].

During the test, the large-screen display was placed at a distance of three meters and the patient’s distance vision was corrected based on refraction. There were 25 trials, during which the QCSF method presented triplets of filtered Sloan letters at 128 contrast levels (0.002 to 100%) and 19 spatial frequencies (approximately 1 to 27 cycles per degree, with higher cycles per degree indicating smaller numbers) with an overall possible 2432 stimuli [7]. The patients were asked to read the letters, and the examiner recorded whether the answer was correct or incorrect or if there was no answer provided. The QCSF device selected the optimal two-dimensional contrast and spatial frequency combinations using a Bayesian Adaptive Algorithm to update the probabilities of the CS curve parameters. This enables the test to have a very high test retest repeatability [23] . The test then calculates the Area Under the Log Contrast Sensitivity Function (AULCSF) using a built in algorithm [25], which integrates the contrast sensitivity at 1.0 to 18 cpd. The AULCSF and the CS at 1.0, 1.5, 3.0, 6.0, 12.0, and 18.0 cpd were recorded for each eye and used for statistical analysis.

### Statistical analysis of data

Data were summarized with means and standard deviations for continuous variables and counts and proportions for categorical variables. Comparisons involving only patient-level (e.g., age, sex) were formally tested with Student’s t-test or Fisher’s Exact Test. Comparisons involving eye-level data (e.g., visual acuity, contrast sensitivity) utilized general estimating equations (GEE) to allow for correlated measurements. Multi-group comparisons (e.g., contrast sensitivity in different diagnosis groups) were made by Wald chi-square tests followed by pairwise comparisons, adjusted for multiple comparisons. Statistical analyses were performed in R (R Foundation for Statistical Computing, Vienna, Austria).

## Results

### Patient characteristics

We included 17 patients (30 eyes) with IRD and 18 (18 eyes) controls. There were 12 patients with rod-cone dystrophy (RCD) caused by pathogenic variants in eight different genes (Table 1), three patients with STGD caused by two pathogenic variants in the *ABCA4* and two patients with Best disease caused by at least one pathogenic variant in the *BEST1.* Four eyes of patients with IRD were excluded because they did not meet visual acuity criteria. Characteristics of the patients with IRD and control group are summarized in Table 1. Patients from the control group were older than patients with IRD (*p* < 0.02) by a mean of 10.7 years but had a similar sex ratio. The control group had a better visual acuity than patients with IRD (*p* < 0.0001). The average visual acuity for the IRD group corresponded to a 20/25 Snellen line, while for the control group it was 20/16. There were no statistically significant differences in visual acuity among the IRD subgroups.

**Table 1:** This table summarizes the patient characteristics for the three dystrophy groups and healthy controls

	Rod-cone dystrophy	Stargardt disease	Best disease	Controls	*P* value IRD vs control
N (%)	12 (22.2%)	3 (5.6%)	2 (3.7%)	18 (52.8%)	NA
Age in years, mean (SD)	36.6 (15.9)	57.0 (13.7)	33.5 (14.4)	52.9 (11.6)	*P* < 0.02^a^
Gender					
Male, n (%)	5 (41.7%)	1 (33.3%)	1 (50.0%)	9, (47.3%)	*P* = 0.74^b^
Female, n (%)	7 (58.3%)	2 (66.7%)	1 (50.0%)	10, (52.6%)	
LogMAR visual acuity, Mean (SD)	0.10 (0.09)	0.18 (0.11)	0.15 (0.17)	−0.10 (0.07)	*P* < 0.0001^c^
Gene mutation, N (%)	*USH2A*, 4 (33%)	*ABCA4* (100%)	*BEST1* (100%)	NA	NA
	*RP1*, 2 (16.7%)				
	*ABHD12*, 1 (8.33%)				
	*RPFF8*, 1 (8.33%)				
	*RHO*, 1 (8.33%)				
	*FLVCR1*, 1 (8.33%)				
	*EYS*, 1 (8.33%)				
	*RPPF31*, 1 (8.33%)				

^a^Student t-test

^b^Fisher Exact Test

^c^Wald test based on GEE model

*NA* not applicable

*IRD* Inherited retinal degeneration

This table summarizes the patient characteristics for the three dystrophy groups and healthy controls

### Contrast sensitivity

Table 2 summarizes the performance on the QCSF for all patient groups. Patients with IRD had lower AULCSF than normal controls (*p* < 0.001). When comparing the CS at different spatial frequencies, we found that patients with IRD had reduced CS at all spatial frequencies (*p* < 0.001) (Table 2). The largest differences were found between 6.0 to 18.0 cpd.

**Table 2:** This table summarizes the contrast sensitivity for the patient groups

	Controls	Rod-cone dystrophy	Best Disease	Stargardt Disease (STGD)	*P*-value dystrophy versus controls	*P* value STGD vs other IRD
AULCSF	1.58 (0.15)	1.27 (0.20)	1.23 (0.32)	0.61 (0.27)	*P* < 0.0001	*P* < 0.001
1.0cpd	1.67 (0.10)	1.50 (0.12)	1.46 (0.20)	1.19 (0.25)	*P* < 0.0001	0.179
1.5 cpd	1.73 (0.10)	1.56 (0.13)	1.50 (0.25)	1.10 (0.22)	*P* < 0.0001	*P* < 0.001
3.0 cpd	1.77 (0.10)	1.54 (0.16)	1.46 (0.29)	0.84 (0.22)	*P* < 0.0001	*P* < 0.0001
6.0 cpd	1.60 (0.17)	1.27 (0.24)	1.23 (0.34)	0.45 (0.36)	*P* < 0.0001	*P* < 0.0001
12.0 cpd	1.08 (0.25)	0.63 (0.31)	0.65 (0.37)	0.19 (0.38)	*P* < 0.0001	*P* = 0.04
18.0 cpd	0.60 (0.31)	0.16 (0.23)	0.22 (0.25)	0.08 (0.17)	*P* < 0.0001	*P* = 1.00

The means (standard deviations) for the AULCSF and contrast sensitivity at each spatial frequency in cycles per degree (cpd) are provided. *P*-values are from Wald test based on GEE model

*AULCSF* Area Under the Log Contrast Sensitivity Function

*IRD* Inherited retinal degeneration

*STGD* Stargardt disease

*Cpd* cycles per degree

*GEE* general estimating equations

This table summarizes the contrast sensitivity for the patient groups. The means (standard deviations) for the AULCSF and contrast sensitivity at each spatial frequency (in cpd) are provided. P-values are from Wald test based on GEE model

**Fig. 1:**
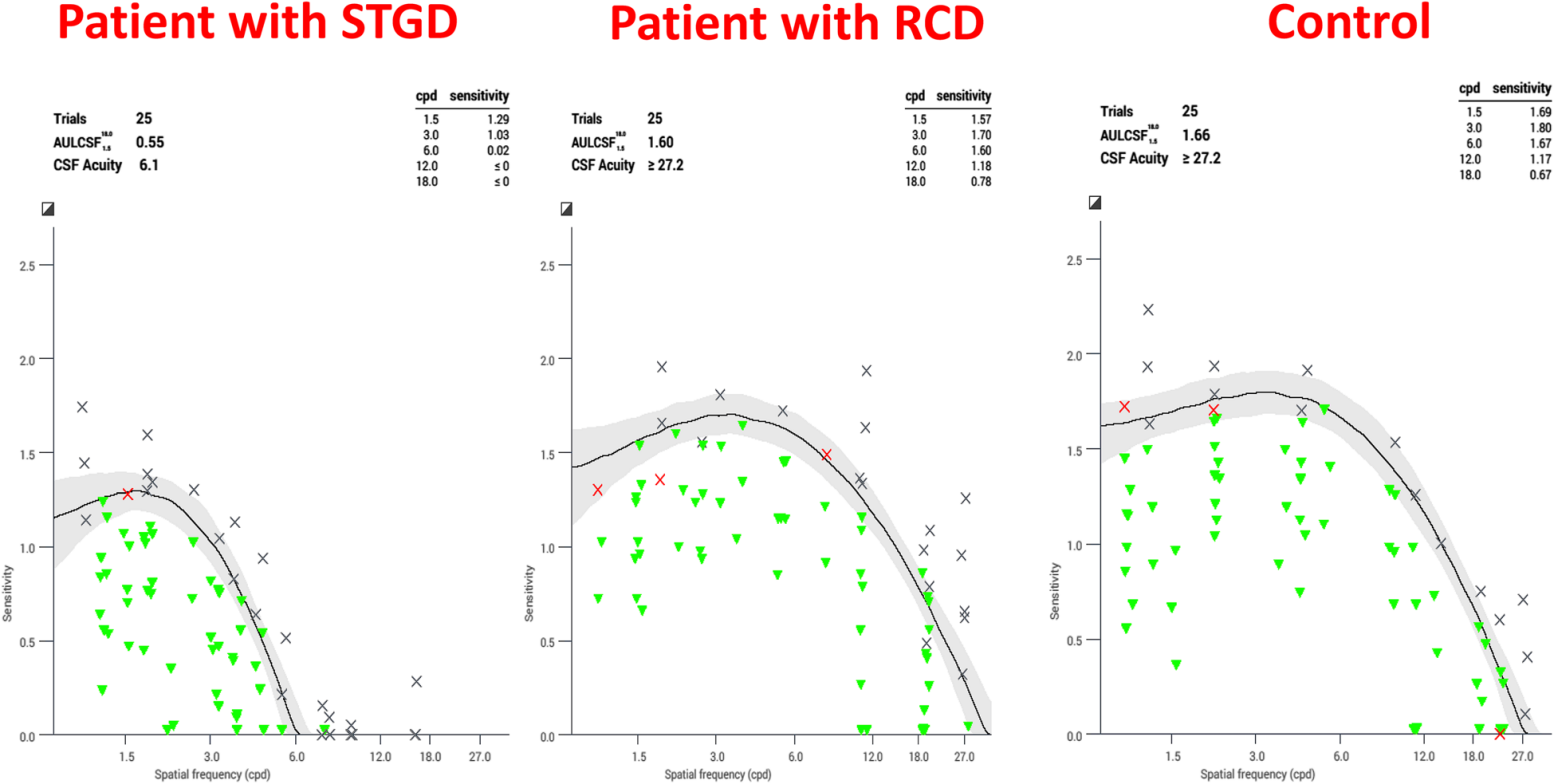
This figure depicts QCSF graphs for age matched subjects with contrast sensitivity on the y axis and spatial frequencies (in cpd) on the x axis. The graph on the left is from a patient with STGD; in the center from a patient with RCD; and on the right is from a control subject. The line is the best fitting contrast sensitivity function with the confidence interval shaded in grey. The green triangles represent the stimuli that the subject captured correctly, the red crosses represent the stimuli that the patient read incorrectly, and the black crosses represent the stimuli that the subject could not read altogether. The patients with STGD had the worst CS function (curve with lower values and smallest area) of all groups. We did not include a CS graph from a patient with Best disease since these patients had an almost identical CS function to patients with RCD

When comparing the different IRD groups, we found that patients with STGD had lower AULCSF than patients with RCD (*p* < 0.001) and Best disease (p < 0.001) (Fig. 1) and significant reductions at all spatial frequencies except 1.0 and 18.0 cpd (Table 2). The greatest differences were found at 3.0 and 6.0 cpd. There were no statistically significant differences in CS between RCD and Best disease. Five patients RCD had CME and 7 did not have it. Patients with CME had an average AULCSF of 1.29, while those without CME had an average AULCSF of 1.25. There were no statistically significant differences in CS in patients with RCD with CME and those without CME (*p* > 0.3). The sample did not allow the analysis of any statistically significant differences in CS among different causative genes.

## Discussion

In this study, we demonstrated that patients with IRD have significant deficits in CS when visual acuity is relatively preserved. By using the QCSF test, we also showed that CS in these patients is reduced in all spatial frequencies. We also found the greatest reductions in CS at the higher spatial frequencies (from 6.0 to 18.0), which is consistent with previous studies [19]. These changes may be missed by Pelli-Robson testing which at fewer spatial frequencies. Furthermore, patients with STGD had worse CS than patients with the other IRDs, even though visual acuities were similar between IRD subgroups. Our study therefore indicates that CS is not merely a surrogate for visual acuity, as it can provide more information about visual dysfunction in these patients than visual acuity alone.

It is not clear why the patients with STGD had a worse CS. Although the patients with STGD were on average older, they were similar in age to control patients, in whom a similarly poor CS was not seen, even though small deteriorations of CS have been associated with aging [26]. This clinical finding for STGD should be considered as future studies unveil the pathogenesis of the disease and the mechanisms for visual dysfunction, such as lipofuscin accumulation in the retinal pigment epithelium, all trans retinal accumulation in the photoreceptors, loss of retinal pigment epithelium (RPE cells), and loss of photoreceptor cells.

Although a variety of studies have measured CS when visual acuity is near 20/20 [7, 27, 28], it is unclear to date what aspect of retinal neuronal function is assessed by CS. It may be that visual acuity only requires a small number of functioning cones in the fovea, while intact CS requires a greater number of intact cones throughout the macula. This may explain why the patients with STGD had a worse CS, as foveal cones may be affected earlier in the disease course [29]. There may also be image processing by inner retinal layers that contribute to CS.

It is also unclear why patients with RCD and CME did not have more alterations in the CS than those without CME. There have been a variety of studies in which CS was reduced in the presence of CME from diseases like diabetic retinopathy or acute central serous retinopathy [28, 30]. It may be that the CS depends on the severity of the edema on OCT. It could also be that CS is dependent on the state of the photoreceptors, and thus the pathology causing both the photoreceptor dysfunction and the edema may be important. Further structural studies using SD-OCT in more patients are needed, so that the severity and duration of CME can also be associated with reductions in CS in IRDs.

Current endpoints for therapeutic trials for STGD use visual acuity (ETDRS) and CS using Pelli-Robson charts to measure central visual function. Since Pelli-Robson charts measure CS at a limited number of spatial frequencies, the magnitude of the visual burden and variations of CS may go undetected in a clinical trial setting that uses only Pelli-Robson as a method to measure CS. Other laboratory methods for assessing CS that use multiple spatial frequencies, such as the VCTS chart, are time-consuming and less practical for use in clinics [23]. The QCSF overcomes some of these inefficiencies and can be used in therapeutic trials as a secondary outcome measure and in clinical practice to demonstrate and monitor unrecognized visual burden from IRD, especially in patients with STGD.

A limitation of our study was the small sample size due to the difficulty of finding patients that met inclusion criteria for the study. It is possible that the specific gene mutations along with the disease severity could affect CS in patients with IRDs, but the sample size is too small to study the effects of different mutations on CS. The small sample size also meant that we could not run a detailed structural analysis of which deficits on SD-OCT were associated with a worse CS in patients with STGD. Due to the sample size, we also cannot make conclusions about CS deficits between patients with STGD and the other mutation proven IRDs. Another limitation for this study is that we did not examine test-re test variability. However, previous studies have reported a very high test retest repeatability with the QCSF [7, 23]. The patients from control group had a better acuity than the patients with IRD. Thus, it is unclear whether the CS would have been worse if the average acuities were the same. However, patients with STGD had similar acuities to the other IRD groups and yet had a worse CS.

## Conclusions

In conclusion, patients with IRD, especially patients with STGD with relatively preserved visual acuity were found to have marked abnormalities in CS when tested across different spatial frequencies. We recommend that clinical trials for STGD incorporate CS tested across different spatial frequencies as a secondary clinical endpoint. Future studies should also correlate the pathogenesis of this disease to the severe losses in CS.

## Abbreviations

AULCSF: Area under the log contrast sensitivity function
CME: Cystoid macular edema
CS: Contrast sensitivity
IRD: Inherited retinal degeneration
QCSF: Quick contrast sensitivity function
RCD: Rod cone dystrophy
SD-OCT: Spectral domain optical coherence tomography
STGD: Stargardt Disease
